# An Updated Review of Emerging Sources of Selenium in Weaned Piglet Nutrition

**DOI:** 10.3390/ani14172599

**Published:** 2024-09-06

**Authors:** Wenyue Zhou, Zheng Yang, Jiajun Han, Xingping Chen, Tiande Zou, Jinming You, Jun Chen

**Affiliations:** Jiangxi Province Key Laboratory of Animal Nutrition and Feed, Jiangxi Province Key Innovation Center of Integration in Production and Education for High-Quality and Safe Livestock and Poultry, Jiangxi Agricultural University, Nanchang 330045, China; 18348392842@163.com (W.Z.); 15737566134@163.com (Z.Y.); 19845198016@163.com (J.H.); cxp0315@jxau.edu.cn (X.C.); tiandezou@jxau.edu.cn (T.Z.); youjinm@163.com (J.Y.)

**Keywords:** anti-inflammation, antioxidant, emerging selenium sources, stress challenges, weaned piglets

## Abstract

**Simple Summary:**

Weaned piglets play a pivotal role in swine production, and they encounter serious stress challenges associated with oxidative stress and inflammation. Selenium (Se) is an indispensable element for pigs, exhibiting well-documented functions that encompass antioxidative and anti-inflammatory properties through the action of selenoproteins. Herein, this review aims to summarize the emerging sources of Se in weaned piglet nutrition over the past decade (from 2013 to the present) to deepen our understanding of emerging Se sources and their practical applications in weaned piglet production.

**Abstract:**

The antioxidant and immune systems of weaned piglets are not fully mature and are also subjected to serious stress challenges related to oxidative stress and inflammation. Selenium (Se) is an essential element for pigs, with documented roles encompassing antioxidative and anti-inflammatory properties via selenoproteins. Sodium selenite and Se-enriched yeast are commonly acknowledged as conventional sources of Se for piglets. In the past decade, several novel Se sources have emerged in the field of weaned piglet nutrition. In this review, we will initially outline the historical timeline of Se sources as reported in weaned piglet nutrition. Afterwards, our attention will turn towards the nutritional regulation of Se sources in relation to the antioxidant and anti-inflammatory aspects of healthy weaned piglets. Ultimately, we will provide a detailed review highlighting the potential of emerging Se sources in alleviating various adverse effects of stress challenges faced by weaned piglets. These challenges include oxidative stress, enterotoxigenic *Escherichia coli* infection, lipopolysaccharide-induced inflammation, heat stress, and exposure to feed mycotoxins. The output of this review will emphasize the fundamental importance of incorporating emerging Se sources in the diet of weaned piglets.

## 1. Introduction

Swine farming is currently the fastest-growing sector within the livestock industry globally, and it is predicted that this prevailing trend will persist for years to come [1]. Pork constitutes over a third of the global meat production, making it a vital element in ensuring global food security and contributing significantly to agricultural economies and international trade [2]. Weaning is widely acknowledged as a critical phase in swine production, with a direct impact on the economic benefits of pig farms [3]. Under natural circumstances, the weaning of piglets typically occurs between the ages of 14 and 18 weeks [4]. It can be defined as a gradual reduction in sow–piglet interaction, along with a decrease in nursing frequency, milk yield, and a gradual shift from sow milk to solid or semisolid feeds [5]. In contrast, under commercial swine farming conditions, weaning typically occurs abruptly at around three to four weeks of age [6]. The antioxidant and immune systems of weaned piglets have yet to reach maturity [7]. It has been demonstrated that weaning leads to the disturbance of redox balance, resulting in oxidative injury in piglets [8,9]. Weaning has also been reported to activate inflammatory responses [10] and to induce the production of inflammatory cytokines in piglets [11]. Importantly, weaned piglets also face numerous exogenous stress challenges that have been well-documented to induce oxidative stress and inflammatory responses. These challenges encompass social factors [12], feed-related factors [13], environmental factors [14], pathogenic factors [15], and others [16]. Consequently, these factors hamper the growth, development, health, and overall production performance of piglets, thereby affecting economic benefits [3].

Selenium (Se) is a vital element for humans and animals, with reported functions including antioxidant and anti-inflammatory properties through the action of selenoproteins [17,18]. Se is an indispensable trace mineral for swine across all stages of their lives [19,20,21]. It plays a crucial role in sow nutrition during gestation and lactation, facilitating the transfer of Se to their fetus and suckling piglets through the placenta and mammary gland [19,22,23]. Additionally, Se influences boar fertility and sperm quality [20], as well as growth and pork quality in growing-finishing pigs [24,25,26]. In particular, this review will primarily focus on emerging Se sources specifically beneficial for weaned piglets. Sodium selenite was first reported as the Se source for weaned piglets [27], followed by the introduction of Se-enriched yeast in their diets [28]. Those two Se sources are widely recognized as conventional Se sources in the field of swine nutrition [22,23]. Over the past decade, several emerging Se sources have been documented in the realm of weaned piglet nutrition, primarily focusing on the modulation of antioxidant and anti-inflammatory mechanisms in piglets. In this review, we will initially present the historical timeline of Se sources reported in the field of weaned piglet nutrition. Subsequently, our focus will shift towards the nutritional regulation of Se sources in relation to the antioxidant and anti-inflammatory aspects of healthy weaned piglets. The summary of emerging selenium sources for healthy weaned piglets over the past decade is provided in Table 1. Finally, we will provide a detailed review highlighting the potential of emerging Se sources in alleviating various adverse effects of stress challenges faced by weaned piglets. These challenges include oxidative stress, enterotoxigenic *Escherichia coli* (ETEC) infection, lipopolysaccharide (LPS)-induced inflammation, heat stress, and feed mycotoxin exposure. The summary of emerging Se sources for stress-challenged weaned piglets over the past decade is present in Table 2.

## 2. Historical Timeline of Se Sources Reported in the Field of Weaned Piglet Nutrition

The significance of Se was not widely recognized in swine nutrition until Eggert et al. established its essentiality as a nutrient in swine by demonstrating its role in preventing hepatosis dietetica [47]. The earliest documented source of Se supplementation in the diets of weaned piglets can be traced back to 1978, with the use of sodium selenite [27]. Since then, sodium selenite has been utilized as a conventional Se source for weaned piglets and remains in practice until the present [48,49]. In 2011, the beneficial effects of Se-enriched yeast on weaned piglets were reported by Zavodnik et al. [28]. Moreover, it has been reported that Se-enriched yeast demonstrates a protective effect against diquat-induced oxidative stress [50], intestinal mucosa disruption [51], and inflammation caused by *Salmonella typhimurium* in weaned piglets [52]. The two Se sources, namely sodium selenite and Se-enriched yeast, are widely recognized as conventional Se sources in the field of swine nutrition [22,23].

**Figure 1:**
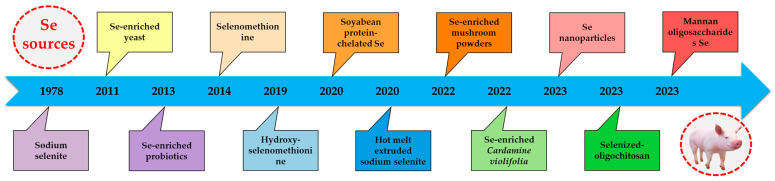
Historical timeline of selenium (Se) sources reported in the field of weaned piglet nutrition for the first time [14,27,28,29,30,31,33,34,37,38,39,45].

Over the past decade, specifically since 2013, several novel Se sources have emerged in the realm of weaned piglet nutrition. In 2013, Gan et al. reported the utilization of Se-rich probiotics in weaned piglet nutrition. Utilizing sodium selenite as the control group, they discovered that Se-enriched probiotics effectively regulated the expression of heat shock proteins of piglets during heat stress conditions [14]. After that, Gan’s research team also documented the impact of Se-enriched probiotics on the regulation of antioxidant and immune functions [43] as well as intestinal microorganisms in heat stress conditions [42]. Selenomethionine and hydroxy-selenomethionine have also been documented as dietary Se sources for antioxidant and immunity of weaned piglets [30,32,36]. Since 2020, seven Se sources in the diets of weaned piglets have been reported successively, including soybean protein-chelated Se [38], hot melt extruded sodium selenite [31], Se-enriched mushroom powders [34], Se-enriched *Cardamine violifolia* [33], Se nanoparticles [37], selenized-oligochitosan [45], and mannan oligosaccharides Se [39]. The historical timeline of Se sources reported in the field of weaned piglet nutrition for the first time is illustrated in Figure 1. In the following section, we will focus on the antioxidant and anti-inflammatory effects of emerging Se sources on healthy weaned piglets over the last decade (2013–present).

## 3. Emerging Se Sources for Healthy Weaned Piglets: Focusing on Antioxidant and Anti-Inflammation Functions

### 3.1. Antioxidant Function

Weaning stress is a great challenge faced by piglets, and oxidative stress is a potential cause of weaning stress in piglets [8,53]. Se is an essential element for pigs primarily because it is involved in the synthesis of 25 selenoproteins that contain selenocysteine residue [54]. Many selenoproteins are classified as oxidoreductases, with the selenocysteine residue having a key role in catalyzing redox regulation and antioxidant activity [55]. According to a study conducted by Rao et al., dietary supplementation of hydroxy-selenomethionine at a dosage of 0.3 mg/kg Se resulted in higher levels of Se in the serum, liver, and muscle of weaned piglets when compared to sodium selenite at the same dosage of 0.3 mg/kg Se [36]. Similar results were reported by Chao et al., where they observed that administering the hydroxy-analogue of selenomethionine at a dosage of 0.3 mg/kg Se resulted in elevated Se levels in the serum, liver, kidney, and muscle of weaned piglets [30]. Chao et al. also demonstrated an enhancement in the antioxidant status of piglets fed hydroxy-analogs of selenomethionine diets, indicated by an increase in serum superoxide dismutase (SOD) activity, a decrease in malondialdehyde (MDA) level, and an increase in hepatic total antioxidant capability (T-AOC) [30]. In another study, selenomethionine, utilized as a source of Se, demonstrated the ability to improve the antioxidant capacity of weaned piglets when compared to sodium selenite. This was evidenced by the notable increase in serum T-AOC and glutathione peroxidase (GSH-Px) activity, in addition to enhanced GSH-Px activity observed in the muscle [29]. The enhanced antioxidant capacity of weaned piglets, such as increased serum SOD activity, was also reported when supplemented with Se-enriched *Cardamine violifolia*, another source of Se, in comparison to Se-enriched yeast [33].

The utilization of hot melt extruded sodium selenite as a dietary Se source in weaned piglets was initially documented in 2020 by Lee et al. [31]. The authors found that the Se content in the liver of weaned piglets was increased through dietary supplementation with hot melt extruded sodium selenite (0.3 mg/kg Se) as compared to dietary supplementation with sodium selenite or selenomethionine at an equivalent dosage of Se [31]. More importantly, the antioxidant status and selenoprotein expression were significantly elevated through the supplementation of hot melt extruded sodium selenite [31]. In comparison to sodium selenite, the supplementation of hot melt extruded sodium selenite resulted in an increase in serum Se level and GSH-Px activity. Moreover, it led to an augmented mRNA expression of *GPx1*, *GPx3*, *GPx4,* and *SELW* in the liver, as well as increased mRNA expression of *GPx1*, *GPx2*, *GPx3*, and *GPx4* in the spleen of the piglets [31]. Given the Se deposit, antioxidant status, and selenoprotein expression, the overall additive effects of the three Se sources for weaned piglets were as follows: hot melt extruded sodium selenite exhibited the highest effect, followed by selenomethionine, and then sodium selenite.

Se nanoparticles, as an emerging source of Se, have been proven to significantly improve intestinal antioxidant status and reduce intestinal oxidative stress in weaned piglets [37]. In a study conducted by Qiao et al., it was observed that the supplementation of Se nanoparticles had a significant positive impact on various antioxidant parameters in piglets [37]. Specifically, there was an increase in jejunal Se level, as well as enhanced jejunal activities of T-AOC, catalase (CAT), total superoxide dismutase (T-SOD), GSH-Px, and thioredoxin reductase (TrxR). Additionally, the study revealed a decrease in jejunal reactive oxygen species (ROS), MDA, and 8-hydroxy-2′-deoxyguanosine (8-OHdG) levels [37]. The transcriptome of jejunal selenoproteins was further analyzed, revealing that the administration of Se nanoparticles resulted in an augmented mRNA expression of *GPX2*, *GPX3*, *GPX4*, *SELENOI*, *SELENOS*, *SEPHS2*, *TXNRD1*, and *TXNRD3* in the jejunum of piglets [37]. Collectively, the antioxidant function serves as the primary foundation for the biological functions of Se, facilitated by the hydroxy-analogue of selenomethionine, selenomethionine, Se-enriched *Cardamine violifolia*, hot melt extruded sodium selenite, and Se nanoparticles in weaned piglets.

### 3.2. Anti-Inflammation Function

Weaning stress is a prevailing concern in piglet production, and it is greatly linked to the inflammatory response process [11]. One of the prominent physiological responses to piglet weaning stress is the activation of the inflammatory response [56]. Implementing nutritional strategies to mitigate intestinal inflammation is a highly effective approach to foster growth and enhance overall health in weaned piglets [57]. A variety of emerging Se sources have been reported to possess anti-inflammatory properties in weaned piglets. Zhang et al. documented that introducing a dietary supplement of 0.3 mg/kg Se in the form of selenomethionine significantly elevated the serum immunoglobulin M level in weaned piglets compared to an equivalent amount of Se provided as sodium selenite [32].

Most recently, the use of Se-enriched mushroom powders as a new source of Se in the regulation of intestinal immunity and health has been reported in weaned piglets [35]. Dowley et al. noted that the introduction of dietary supplementation with 0.3 mg/kg Se through Se-enriched mushroom powders resulted in improved intestinal immunity in weaned piglets [35]. This improvement was demonstrated by a notable reduction in the mRNA expression of *IL-10* and *TLR4* in the duodenum [35]. The authors also found that supplementing Se-enriched mushroom powders resulted in an increased abundance of *Lactobacillaceae* and *Roseburia* in the cecal digesta of piglets [35]. Additionally, the incorporation of Se-enriched mushroom powders has been found to improve the intestinal morphology and nutrient transport of weaned piglets. This is indicated by the increased jejunal villous height (VH) and villous height/crypt depth (VH/CD), ileal VH, and elevated jejunal *FABP2* mRNA expression [35]. Conway et al. also conducted research on the combined supplementation of Se-enriched mushroom powders and sodium selenite (0.3 + 0.3 mg/kg) for weaned piglets in a 39-day feeding trial [34]. The researchers discovered that the combined supplementation significantly enhanced the growth performance of piglets throughout the feeding trial from day 21 to 39 and also discovered elevated Se deposits in the liver and muscle of the piglets at day 39 of the study [34]. Additionally, the concurrent supplementation exhibited an augmentation in the jejunal mRNA levels of *SGLT1* and *SELENOP*, suggestive of an elevation in intestinal nutrient transport [34]. Regarding gut microbiota, the combined supplementation resulted in an increase in the abundance of *Prevotella*, *Prevotellamassilia*, and *Faecalibacterium*, and a decrease in the abundance of *Clostridium*, *Sporobacter*, and *Ruminococcus* in the cecal digesta of piglets [34].

Se nanoparticles, an emerging Se source, have been reported as a novel approach for addressing diarrhea and promoting growth performance in weaned piglets [37]. These nanoparticles effectively regulate the intestinal inflammation of piglets over the course of the two-week post-weaning period [37]. Dietary supplementation with 0.3 mg/kg of Se in the form of Se nanoparticles has been shown to significantly enhance average daily gain (ADG) and average daily feed intake (ADFI) while reducing the feed conversion ratio (FCR) and diarrhea incidence in weaned piglets [37]. The administration of Se nanoparticles resulted in an elevation of serum Se, albumin, secretory immunoglobulin A (sIgA), and transforming growth factor-β (TGF-β) levels, while simultaneously reducing serum diamine oxidase (DAO), D-lactic acid, LPS, and interleukin-18 (IL-18) levels in piglets [37]. Regarding intestinal inflammation, the addition of Se nanoparticles resulted in an increase in jejunal sIgA and TGF-β levels, as well as a decrease in jejunal interleukin-1β (IL-1β), IL-18 and tumor necrosis factor-α (TNF-α) contents [37]. The presence of Se nanoparticles resulted in an increase in both the mRNA and protein expression of jejunal MUC2 in piglets. The cecal microbiota and short-chain fatty acids (SCFA) levels are regulated by Se nanoparticles, which have been found to increase the abundance of *Holdemanella* and the levels of acetate, propionate, and total SCFA in the cecal digesta of weaned piglets [37]. Together, the anti-inflammatory function is another key biological characteristic of Se. Se nanoparticles, selenomethionine, and Se-enriched mushroom powders can be utilized as a Se source for regulating inflammation in weaned piglets.

## 4. Emerging Se Sources for Stress-Challenged Weaned Piglets

### 4.1. Oxidative Stress Challenge

Oxidative stress is a significant indicator of weaning stress in piglets and has adverse effects on their growth performance and overall health [58]. Additionally, improper management practices, environmental stressors, feed oxidation, and pathogen infections have all been documented as factors that can induce or worsen oxidative damage in weaned piglets [38]. In an experimental model of diquat-induced oxidative stress, Se-enriched yeast was found to mitigate growth retardation in weaned piglets [50]. This effect was attributed to the augmentation of antioxidant capacity and immune functions, as well as the suppression of inflammatory response [50]. An emerging Se source, soybean protein-chelated Se, was also assessed for its mitigating effects on diquat-induced oxidative stress in weaned piglets [38]. Dietary supplementation with either 0.3 mg/kg Se as soybean protein-chelated Se or selenized yeast partially mitigated oxidative stress in weaned piglets, as observed in a diquat-induced oxidative stress model [38]. Specifically, dietary supplementation of soybean protein-chelated Se increased SOD activity in the plasma of piglets experiencing oxidative stress [38]. Moreover, the dietary supplementation of selenized yeast showed improvement in antioxidant capacity and immune function, as demonstrated by increased GSH-Px activity in both plasma and liver, as well as a decrease in lymphocyte count in the blood of piglets [38]. According to the findings of Doan et al., the additive effects of the evaluated Se sources under diquat-induced oxidative stress conditions are ranked as follows: selenized yeast > soybean protein-chelated Se > sodium selenite [38].

### 4.2. ETEC Infection Challenge

Aside from oxidative stress, which has also been reported to be induced by ETEC infection in piglets [59], pathogenic infection presents a prominent challenge that negatively affects the growth and intestinal health of weaned piglets [60]. Of note, ETEC stands out as the primary contributor to postweaning diarrhea in piglets, representing a prominent challenge within piglet production [61]. The occurrence of ETEC infection often leads to intestinal barrier dysfunction, intestinal inflammation, and gut microbiota imbalance and potentially results in the morbidity and mortality of weaned piglets [62]. Importantly, a novel source of Se, mannan oligosaccharides Se (MOSS), has been reported to effectively alleviate the diarrhea caused by ETEC in weaned piglets [39]. Specifically, dietary supplementation with 0.4 mg/kg MOSS reduced the diarrhea frequency and diarrhea index and increased the ADG of piglets with ETEC infection [39]. Also, MOSS supplementation was observed to enhance antioxidant and immune functions in weaned piglets. This was supported by an increase in ileal T-AOC and GSH levels, a decrease in blood neutrophil count and serum C-reactive protein, and a reduction in ileal interleukin 17 (IL-17) levels [39]. Regarding intestinal integrity, MOSS supplementation led to a reduction in levels of serum DAO, D-lactate, endotoxin, and an increase in protein expression of ZO-1 in both the jejunum and ileum. Additionally, there was a decrease in jejunal and ileal crypt depth (CD), accompanied by an elevation in jejunal and ileal VH/CD in piglets [39]. Herein, the utilization of MOSS as a promising Se source for weaned piglets, who potentially suffer from ETEC infection, could be considered.

### 4.3. LPS-Induced Inflammation Challenge

LPS is an endotoxin found in Gram-negative bacteria (including ETEC) that has the ability to stimulate excessive production of pro-inflammatory cytokines, leading to the development of inflammation [63]. Se-enriched *Cardamine violifolia*, which has recently been identified as a dietary Se source in pigs, demonstrated the ability to effectively alleviate liver damage and inflammation induced by LPS in weaned piglets [40]. Dietary supplementation with 0.3 mg/kg Se from Se-enriched *Cardamine violifolia* resulted in decreased serum aspartate aminotransferase (AST) and alkaline phosphatase (ALP) levels, as well as an increase in hepatic GSH-Px activity and a decrease in MDA levels in LPS-challenged piglets [40]. With regards to inflammation, the administration of Se-enriched *Cardamine violifolia* resulted in a decrease in hepatic concentrations of interleukin 6 (IL-6) and TNF-α, along with a reduction in hepatic mRNA levels of *IL-6*, *TNF-α*, *MyD88*, *NOD1*, *RIPK2* and *COX2* in piglets exposed to LPS challenge [40]. Additionally, the supplementation of Se-enriched *Cardamine violifolia* led to a reduction in both the mRNA level and protein level of RIPK1, RIPK3, and MLKL in the liver of piglets [40].

Se-enriched *Cardamine violifolia* was also reported to mitigate intestinal injury induced by LPS challenge in weaned piglets [41]. Dietary addition with Se-enriched *Cardamine violifolia* improved the jejunal morphology, jejunal enzyme activities, and jejunal barrier function of LPS-challenged piglets [41]. These improvements were indicated by increased VH, VH/CD, lactase activity, and maltase activity, as well as enhanced protein expression of Occludin and ZO-1 in the jejunum [41]. Additionally, the supplementation of Se-enriched *Cardamine violifolia* demonstrated improvements in antioxidant function and immunity. This was indicated by elevated T-AOC and SOD activity and reduced IL-6 levels in the serum [41]. Furthermore, increased SOD activity and decreased *IL-6* mRNA levels were observed in the jejunum of piglets [41]. The elevated antioxidant and immunity can be attributed to the expression of selenoproteins, such as the elevated mRNA levels of *TXNRD1*, *SELENOS*, *SELENOO*, *SEPHS2*, *GPX1*, and *GPX3* in the jejunum of piglets fed Se-enriched *Cardamine violifolia* diets [41]. The potential beneficial effects of Se-enriched *Cardamine violifolia* on LPS-induced intestinal damage in piglets can be associated with the regulation of mitochondrial fusion. This is indicated by the higher levels of protein expression of MFN1, MFN2, and OPA1 observed in the jejunum [41]. Therefore, Se-enriched *Cardamine violifolia* can be considered as a potential Se source to alleviate the liver and intestinal injury induced by LPS in weaned piglets.

### 4.4. Heat Stress Challenge

Heat stress is an additional factor that detrimentally impacts the growth performance and overall health of weaned piglets [64,65]. The adverse effects of heat stress on weaned piglets were mitigated by dietary supplementation with Se-enriched probiotics [14,42,43]. In a study conducted by Lv et al., it was found that dietary addition of 0.3 mg/kg Se in the form of Se-enriched probiotics resulted in improved growth performance of weaned piglets in high ambient temperature conditions [42]. This was indicated by an increase in ADG, a decrease in FCR, and a reduction in the incidence of diarrhea [42]. The authors additionally observed that Se-enriched probiotics supplementation led to an increase in whole blood Se level, GSH-Px activity, and serum triiodothyronine (T3) level and a decrease in serum thyroxine (T4) level in piglets [42]. The fecal microbiota of piglets was regulated by Se-enriched probiotics, resulting in an increased abundance of *Lactobacillus* and a decreased abundance of *Escherichia coli* [42].

Gan et al. conducted a study to assess the impact of dietary inclusion of Se-enriched probiotics on the mRNA expression of heat shock proteins in weaned piglets exposed to heat stress conditions [14]. The authors found that the mRNA expression of *Hsp27* and *Hsp70* were down-regulated in the liver, kidney, and spleen in piglets provided with Se-enriched probiotic supplementation [14]. Concurrently, the supplementation of Se-enriched probiotics resulted in increased Se content in different body parts such as the whole blood, liver, kidney, muscle, and spleen of piglets [14]. Moreover, it also led to increased glutathione peroxidase 1 (GPx1) activity in the erythrocytes, liver, kidney, and spleen, alongside an up-regulation in mRNA expression of *GPx1* in the liver, kidney, and spleen of piglets [14].

The antioxidant and immune functions were also evaluated in heat-stressed weaned piglets fed Se-enriched probiotics-supplemented diets [43]. This study found that the dietary addition of Se-enriched probiotics (0.3 mg/kg Se) resulted in an increase in whole blood GSH-Px activity, erythrocytes GSH level, and serum interleukin 2 (IL-2) level while reducing the serum MDA level in heat-stressed piglets [43]. The elevated antioxidant status was further validated by the up-regulated mRNA level of *TR1* in the liver, kidney, and spleen of piglets [43]. Also, the supplementation of Se-enriched probiotics significantly promoted T lymphocyte proliferation, as evidenced by increased peripheral blood lymphocytes and splenocytes observed in heat-stressed piglets [43]. Finally, the growth performance of weaned piglets was significantly improved, with increased ADG and reduced FCR, when they were fed diets supplemented with Se-enriched probiotics in the presence of heat stress conditions [43]. Based on the results mentioned above, Se-enriched probiotics have shown promise as a novel source of Se to effectively mitigate the compromise in weaned piglets caused by heat stress rearing conditions.

### 4.5. Feed Mycotoxin Challenge

Pigs are highly susceptible to mycotoxins, and their extensive consumption of cereals puts them at risk of chronic contamination [66,67,68]. Mycotoxins cause economic losses in the pig industry, particularly during post-weaning stage when piglets are still physiologically immature [69]. Gan et al. conducted an evaluation of the protective effects of Se-enriched probiotics on kidney impairment in weaned piglets that were fed diets contaminated with ochratoxin A [44]. The researchers discovered that dietary supplementation of 0.3 mg/kg Se as Se-enriched probiotics had a positive impact on the growth performance of weaned piglets fed ochratoxin A-contaminated diets. This was evidenced by increased ADG and ADFI of piglets during the feeding trial period [44]. Additionally, the dietary supplementation of Se-enriched probiotics significantly enhanced serum GSH-Px and SOD activities, while contributing to a decrease in serum creatinine, urea, and MDA levels in piglets [44]. The kidney serves as the primary organ affected by ochratoxin A, a highly potent nephrotoxin [70]. Importantly, the administration of Se-enriched probiotics as a dietary supplement resulted in a significant increase in the mRNA expression of *GPx1*, *GPx4*, *SelS*, *TR1*, and *SOCS3* genes while simultaneously reducing the mRNA expression of *DNMT1* in the kidney of weaned piglets [70]. Additionally, the protein expression of GPx1 and SOCS3 were increased, while the protein expression of DNMT1 was reduced in the kidneys of piglets fed diets supplemented with Se-enriched probiotics [70].

Selenized-oligochitosan, an additional Se source, has been documented to effectively alleviate zearalenone-induced intestinal dysfunction in weaned piglets [45]. The authors found that dietary supplementation of 0.5 mg/kg Se as selenized-oligochitosan exhibited positive effects on the ileal morphology of weaned piglets, including improvements in VH, CD, and VH/CD [45]. Additionally, the supplementation of selenized-oligochitosan resulted in the increased activities of digestive enzymes of piglets, such as trypsin, lipase, and α-amylase, in the ileum [45]. Moreover, the supplementation of selenized-oligochitosan showed positive effects on the intestinal barrier function of piglets. These effects were observed in weaned piglets exposed to zearalenone, as indicated by the increased mRNA expression of *ZO-1*, *Occludin*, and *Claudin-1*, elevated plasma D-xylose levels, and decreased plasma levels of D-lactate and DAO [45].

**Figure 2:**
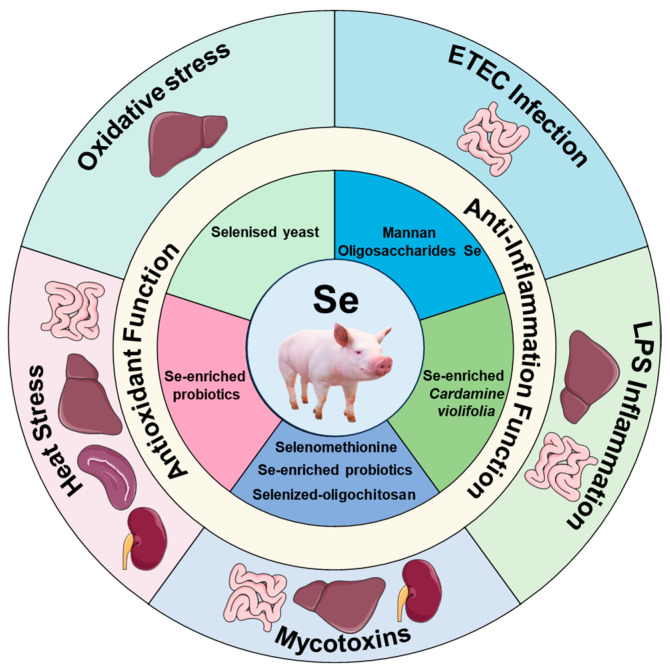
The schematic diagram of emerging selenium (Se) sources for weaned piglets to combat external stress challenges based on current knowledge [14,38,39,40,41,42,43,44,45,46].

The protective effects of selenomethionine against deoxynivalenol-induced liver toxicity were exemplified in weaned piglets [46]. The dietary supplementation with 0.5 mg/kg of Se as selenomethionine resulted in a reduction in the serum AST and lactate dehydrogenase (LDH) activities of the piglets [46]. These activities are considered crucial indicators of hepatic function [46]. Also, the inclusion of selenomethionine in the diet positively influenced hepatic antioxidant status in piglets fed deoxynivalenol-contaminated diets. This was indicated by a reduction in ROS level and an increase in T-AOC, CAT, and GSH-Px activities in the liver, as observed [46]. The study also found that dietary supplementation with selenomethionine led to the reduced hepatic mRNA expression of *NRF-1*, *Bax*, and *CASP9*, while it increased the hepatic mRNA expression of *Gclm*, *NQO1 and GPX1* [46]. Moreover, there was an observed increase in hepatic protein expression of p-JNK in piglets [46]. In light of the aforementioned findings, there is a potential opportunity to mitigate the negative impact of mycotoxin-induced issues in weaned piglets by introducing Se supplements into their diets. Possible interventions may involve the inclusion of Se-enriched probiotics, selenized-oligochitosan, and selenomethionine. The schematic diagram in Figure 2 illustrates the emerging sources of selenium (Se) for weaned piglets, as per the current knowledge, to combat external stress challenges.

## 5. Conclusions

As an essential trace element, Se exerts potent antioxidant and anti-inflammatory functions for weaned piglets through the action of selenoproteins. In light of current findings, emerging Se sources have the potential to be effectively utilized in weaned piglets. Several studies have revealed that several emerging Se sources as a potential defense against oxidative stress, heat stress, mycotoxin toxicity, ETEC infection, or LPS challenge in weaned piglets are highly promising.

## 6. Perspectives

For future research, another pressing challenge is the prohibition of in-feed antibiotics in the swine industry. Traditionally, in-feed antibiotics have been utilized as growth promoters and antimicrobial agents for weaned piglets [71]. However, the utilization of in-feed antibiotics has been banned in various countries and regions, including the European Union, the United States, and China, among others, due to the emergence and development of antimicrobial resistance [72]. In addition, the use of high-dose zinc oxide (at pharmacological levels) as a growth promoter and antimicrobial agent has been prohibited in the diets of weaned piglets in several countries and regions, including the European Union, South Korea, and Japan [73]. Therefore, in the context of the prohibition of in-feed antibiotics and the restriction of high-dose zinc oxide, enhancing the antioxidant and anti-inflammatory functions of weaned piglets via nutritional strategies has become an urgent task in the field of weaned piglet nutrition research. The potential involvement of these emerging selenium sources in these fields and their mechanisms of action require further investigation. In addition to these potential effects, it is imperative to highlight that when assessing the optimal Se supplementation, it is imperative to not only analyze its impacts on growth and diarrhea phenotypes but also prioritize careful attention to the antioxidant and anti-inflammatory attributes of the Se source and its dietary dosage. More importantly, the inclusion of selenoprotein transcriptome as an evaluation criterion should be carefully considered to determine the optimal dietary Se supplementation for weaned piglets.

## Figures and Tables

**Table 1:** The summary of emerging selenium (Se) sources for healthy weaned piglets over the past decade.

Subject	Se Source	Feeding Duration	Main Findings	References
Weaned piglets (average BW: 9.94 kg)	Source (Se level): sodium selenite (0.3 mg/kg Se), selenomethionine (0.1, 0.3, 0.5, 0.7 mg/kg) Recommendation: selenomethionine (0.3 mg/kg)	42 days	Serum antioxidant status: ↑ T-AOC, GSH-Px activity; Muscle antioxidant status: ↑ GSH-Px activity.	[29]
Weaned piglets (average BW: 7.72 kg)	Source (Se level): sodium selenite (0.3 mg/kg Se), hydroxy-analogue of selenomethionine (0.1, 0.2, 0.3, 0.4, 0.5 mg/kg) Recommendation: hydroxy-analogue of selenomethionine (0.3 mg/kg)	28 days	Se content: ↑ serum, liver, kidney, muscle; Serum antioxidant status: ↑ SOD activity, ↓ MDA level; Liver antioxidant status: ↑ T-AOC.	[30]
Weaned piglets (average BW: 6.87 kg)	Source (Se level): sodium selenite (0.3 mg/kg), selenomethionine (0.3 mg/kg), hot melt extruded sodium selenite (0.3 mg/kg) Recommendation: hot melt extruded sodium selenite (0.3 mg/kg)	28 days	Se content: ↑ serum, liver; Serum antioxidant status: ↑ GPx activity; Liver selenoprotein mRNA expression: ↑ *GPx1*, *GPx3*, *GPx4*, *SELW*; Spleen selenoprotein mRNA expression: ↑ *GPx1*, *GPx2*, *GPx3*, *GPx4*.	[31]
Weaned piglets (average BW: 7.6 kg)	Source (Se level): sodium selenite (0.3 mg/kg), Se-enriched yeast (0.3 mg/kg), selenomethionine (0.3 mg/kg) Recommendation: selenomethionine (0.3 mg/kg)	28 days	Serum immuneglobulin levels: ↑ IgM level.	[32]
Weaned piglets (average BW: 9.70 kg)	Source (Se level): sodium selenite (0.3 mg/kg), Se-enriched yeast (0.3 mg/kg), Se-enriched *Cardamine violifolia* (0.3 mg/kg), Se-enriched yeast + Se-enriched *Cardamine violifolia* (0.3 + 0.3 mg/kg) Recommendation: Se-enriched *Cardamine violifolia* (0.3 mg/kg)	28 days	Overall additive effects: Se-enriched *Cardamine violifolia* (0.3 mg/kg) ≈ Se-enriched yeast + Se-enriched *Cardamine violifolia* (0.3 + 0.3 mg/kg); Growth performance: ↓ FCR; Serum antioxidant status: ↑ SOD activity; Jejunal morphology: ↑ VH, VH/CD; Jejunal enzyme activities: maltase, lactase; Jejunal mRNA expression: ↑ *SELENOO*, *SELENOS*, *ZO-1*.	[33]
Weaned piglets (average BW: 6.7 kg)	Source (Se level): sodium selenite (0.3 mg/kg), Se-enriched mushroom powders (0.15, 0.3 mg/kg), selenite + Se-enriched mushroom powders (0.3 + 0.3 mg/kg) Recommendation: selenite + Se-enriched mushroom powders (0.3 + 0.3 mg/kg)	39 days	Growth performance: ↑ ADG, ↓ FCR (day 21–39); Se content: ↑ liver, muscle; Jejunal mRNA expression: ↑ *SGLT1*, *SELENOP*; Cecal microbiota: ↑ *Prevotella*, *Prevotellamassilia*, *Faecalibacterium*, ↓ *Clostridium*, *Sporobacter*, *Ruminococcus*.	[34]
Weaned piglets (average BW: 6.8 kg)	Source (Se level): sodium selenite (0.3 mg/kg), Se-enriched mushroom powders (0.3 mg/kg) Recommendation: Se-enriched mushroom powders (0.3 mg/kg)	39 days	Duodenum mRNA expression: ↓ *IL-10*, *TLR4*; Jejunal mRNA expression: ↑ *FABP2*; Jejunal morphology: ↑ VH, VH/CD; Ileal morphology: ↑ VH; Cecal microbiota: ↑ *Lactobacillaceae*, *Roseburia*.	[35]
Weaned piglets (average BW: 6.0 kg)	Source (Se level): sodium selenite (0.3 mg/kg), Se-enriched yeast (0.3 mg/kg), hydroxy-selenomethionine (0.3 mg/kg) Recommendation: hydroxy-selenomethionine (0.3 mg/kg)	35 days	Se content: ↑ serum, liver, muscle.	[36]
Weaned piglets (average BW: 5.56 kg)	Source (Se level): sodium selenite (0.3 mg/kg), Se nanoparticles (0.3 mg/kg) Recommendation: Se nanoparticles (0.3 mg/kg)	14 days	Growth performance: ↑ ADG, ADFI, ↓ FCR, diarrhea incidence; Jejunal morphology: ↑ VH, goblet cell counts; Serum parameters: ↑ Se, albumin, sIgA, TGF-β levels, ↓ DAO, D-lactic acid, LPS, IL-18 levels; Jejunal Se and antioxidant status: ↑ Se level, T-AOC, CAT, T-SOD, GPx, TrxR activities, ↓ ROS, MDA, 8-OHdG levels; Jejunal immune parameters: ↑ sIgA, TGF-β, ↓ IL-1β, IL-18, TNF-α levels; Jejunal mRNA expression: ↑ *MUC2*, *GPX2*, *GPX3*, *GPX4*, *SELENOI*, *SELENOS*, *SEPHS2*, *TXNRD1*, *TXNRD3*, *TFAM*, *POLG2*; Jejunal protein expression: ↑ MUC2, Claudin-1, Occludin, ZO-1; Cecal microbiota: ↑ *Holdemanella*; Cecal SCFA levels: ↑ acetate, propionate, total SCFA.	[37]

Abbreviations: ↑, increase; ↓, decrease; ADFI, average daily feed intake; ADG, average daily gain; BW, body weight; CAT, catalase; DAO, diamine oxidase; 8-OHdG, 8-hydroxy-2′-deoxyguanosine; *FABP2*, fatty acid binding protein 2; FCR, feed conversion ratio; GPx or GSH-Px, glutathione peroxidase; *GPx1*, glutathione peroxidase 1; *GPx2* or *GPX2*, glutathione peroxidase 2; *GPx3* or *GPX3*, glutathione peroxidase 3; *GPx4* or *GPX4*, glutathione peroxidase 4; IgM, immunoglobulin M; IL-1β, interleukin-1β; *IL-10*, interleukin 10; IL-18, interleukin-18; LPS, lipopolysaccharide; MDA, malondialdehyde; *MUC2*, mucin 2; *POLG2*, DNA polymerase gamma 2; ROS, reactive oxygen species; SCFA, short-chain fatty acids; *SELENOI*, selenoprotein I; *SELENOO*, selenoprotein O; *SELENOP*, selenoprotein P; *SELENOS*, selenoprotein S; *SELW*, selenoprotein W; *SEPHS2*, selenophosphate synthetase 2; *SGLT1*, sodium-glucose cotransporter 1; sIgA, secretory immunoglobulin A; SOD, superoxide dismutase; T-AOC, total antioxidant capability; *TFAM*, mitochondrial transcription factor A; TGF-β, transforming growth factor-β; *TLR4*, toll like receptor 4; TNF-α, tumor necrosis factor-α; TrxR, thioredoxin reductase; T-SOD, total superoxide dismutase; *TXNRD1*, thioredoxin reductase 1; *TXNRD3*, thioredoxin reductase 3; VH, villous height; VH/CD, villous height/crypt depth; *ZO-1*, zonula occludens-1.

**Table 2:** The summary of emerging selenium (Se) sources for stress-challenged weaned piglets over the past decade.

Subject	Stress Model	Se Source	Feeding Duration	Main Findings	References
Weaned piglets (average BW: 9.72 kg)	Diquat	Source (Se level): sodium selenite (0.3 mg/kg), selenized yeast (0.3 mg/kg), soybean protein-chelated Se (0.3 mg/kg) Recommendation: selenized yeast (0.3 mg/kg)	17 days	Overall additive effects: selenized yeast > soybean protein-chelated Se > sodium selenite; Plasma antioxidant status: ↑ GPx activity; Liver antioxidant status: ↑ GPx activity; Leucocyte profile: ↓ lymphocyte count.	[38]
Weaned piglets (average BW: 6.69 kg)	Enterotoxigenic *Escherichia coli*	Source: mannan oligosaccharides Se (MOSS) (0.4 mg/kg MOSS, and the Se content of MOSS is 8.81%) Recommendation: MOSS (0.4 mg/kg)	25 days	Growth performance: ↑ ADG, ↓ diarrhea frequency, diarrhea index; Blood parameters: ↓ neutrophil count, serum C-reactive protein, DAO, D-lactate, endotoxin levels; Ileal antioxidant status: ↑ T-AOC, GSH level; Ileal inflammatory factors: ↓ IL-17 level; Jejunal and ileal morphology: ↓ CD, ↑ VH/CD; Jejunal and ileal protein expression: ↑ ZO-1.	[39]
Weaned piglets (average BW: 9.96 kg)	Lipopolysaccharide	Source (Se level): sodium selenite (0.3 mg/kg), Se-enriched *Cardamine violifolia* (0.3 mg/kg) Recommendation: Se-enriched *Cardamine violifolia* (0.3 mg/kg)	28 days	Serum parameters: ↓ AST, ALP levels; Liver antioxidant status: ↑ GSH-Px activity, ↓ MDA level; Liver cytokine levels: ↓ IL-6, TNF-α; Liver mRNA expression: ↓ *IL-6*, *TNF-α*, *GPX3*, *SELENOI*, *MyD88*, *NOD1*, *RIPK2*, *COX2*, *RIPK1*, *RIPK3*, *MLKL*; Liver protein expression: ↓ RIPK1, RIPK3, MLKL.	[40]
Weaned piglets (average BW: 9.96 kg)	Lipopolysaccharide	Source (Se level): sodium selenite (0.3 mg/kg), Se-enriched *Cardamine violifolia* (0.3 mg/kg) Recommendation: Se-enriched *Cardamine violifolia* (0.3 mg/kg)	28 days	Jejunal morphology: ↑ VH, VH/CD; Jejunal enzyme activities: ↑ lactase, maltase; Plasma parameters: ↑ T-AOC, SOD activity, ↓ IL-6 levels; Jejunal antioxidant status: ↑ SOD activity; Jejunal mRNA expression: ↓ *IL-6*, ↑ *TXNRD1*, *SELENOS*, *SELENOO*, *SEPHS2*, *GPX1*, *GPX3*; Jejunal protein expression: ↑ Occludin, ZO-1, MFN1, MFN2, OPA1.	[41]
Weaned piglets (average BW: 7.9 kg)	Heat stress	Source (Se level): sodium selenite (0.3 mg/kg), Se-enriched probiotics (0.3 mg/kg) Recommendation: Se-enriched probiotics (0.3 mg/kg)	42 days	Growth performance: ↑ ADG, ↓ FCR, diarrhea incidence; Blood parameters: ↑ whole blood Se level, GPX activity, ↑ serum T3 level, ↓ T4 level; Fecal microbiota: ↑ *Lactobacillus*, ↓ *Escherichia coli*.	[42]
Weaned piglets (average BW: 7.9 kg)	Heat stress	Source (Se level): sodium selenite (0.3 mg/kg), Se-enriched probiotics (0.3 mg/kg) Recommendation: Se-enriched probiotics (0.3 mg/kg)	42 days	Se content: ↑ whole blood, liver, kidney, muscle, spleen; Tissue GPx1 activity: ↑ erythrocyte, liver, kidney, spleen; Tissue *Gpx1* mRNA expression: ↑ liver, kidney, spleen; Tissue *Hsp70* mRNA expression: ↓ liver, kidney, spleen; Tissue *Hsp27* mRNA expression: ↓ liver, kidney, spleen.	[14]
Weaned piglets (average BW: 7.9 kg)	Heat stress	Source (Se level): sodium selenite (0.3 mg/kg), Se-enriched probiotics (0.3 mg/kg) Recommendation: Se-enriched probiotics (0.3 mg/kg)	42 days	Growth performance: ↑ ADG, ↓ FCR; Blood antioxidant and immune status: ↑ whole blood GPX activity, erythrocyte GSH level, ↓ serum MDA level, ↑ serum IL-2 level; TCR-induced T lymphocyte proliferation: ↑ peripheral blood lymphocytes, splenocytes; Tissue *TR1* mRNA expression: ↑ liver, kidney, spleen.	[43]
Weaned piglets (average BW: 8.26 kg)	Ochratoxin A	Source (Se level): Se-enriched probiotics (0.15, 0.3 mg/kg) Recommendation: Se-enriched probiotics (0.3 mg/kg)	42 days	Growth performance: ↑ ADG, ADFI; Serum parameters: ↓ creatinine, urea, MDA levels, ↑ GPX, SOD activities; Kidney mRNA expression: ↑ *GPx1*, *GPx4*, *SelS*, *TR1*, *SOCS3*, ↓ *DNMT 1*; Kidney protein expression: ↑ GPx1, SOCS3, ↓ DNMT 1.	[44]
Weaned piglets (Age: 35 days old)	Zearalenone	Source (Se level): selenized-oligochitosan (0.3, 0.5 mg/kg) Recommendation: selenized-oligochitosan (0.5 mg/kg)	42 days	Ileal morphology: ↑ VH, ↓ CD, ↑ VH/CD; Serum parameters: ↓ plasma D-lactate, DAO, ↑ D-xylose levels; Ileal digestive enzyme activities: ↑ trypsin, lipase, α-amylase; Ileal tight junction protein mRNA expression: ↑ *ZO-1*, *Occludin*, *Claudin-1*.	[45]
Weaned piglets (average BW: 6.78 kg)	Deoxynivalenol	Source (Se level): selenomethionine (0.3, 0.5 mg/kg) Recommendation: selenomethionine (0.5 mg/kg)	28 days	Serum parameters: ↓ AST, LDH activities; Liver antioxidant status: ↓ ROS level, ↑ T-AOC, CAT, GSH-Px activities; Liver mRNA expression: ↓ *NRF-1*, *Bax*, *CASP9*, ↑ *Gclm*, *NQO1*, *GPX1*; Liver protein expression: ↓ p-JNK.	[46]

Abbreviations: ↑, increase; ↓, decrease; ADFI, average daily feed intake; ADG, average daily gain; ALP, alkaline phosphatase; AST, aspartate aminotransferase; *Bax*, Bcl2 associated X; BW, body weight; *CASP9*, caspase 9; CAT, catalase; CD, crypt depth; *COX2*, cytochrome c oxidase subunit II; DAO, diamine oxidase; *DNMT 1*, DNA methyltransferase 1; FCR, feed conversion ratio; *Gclm*, glutamate-cysteine ligas modifier subunit; GPx, GPX or GSH-Px, glutathione peroxidase; *Gpx1*, *GPx1*, or *GPX1*, glutathione peroxidase 1; *GPX3*, glutathione peroxidase 3; *GPx4*, glutathione peroxidase 4; GSH, glutathione; *Hsp27*, heat shock protein 27; *Hsp70*, heat shock protein 70; IL-2, interleukin 2; *IL-6*, interleukin 6; IL-17, interleukin 17; LDH, lactate dehydrogenase; MDA, malondialdehyde; MFN1, GTPases mitofusin 1; MFN2, GTPases mitofusin 2; *MLKL*, mixed-lineage kinase domain-like; *MyD88*, myeloid differentiation factor 88; *NOD1*, nucleotide-binding oligomerization domain proteins 1; *NQO1*, NAD(P)H quinone oxidoreductase 1; *NRF-1*, nuclear respiratory factor 1; OPA1, optic atrophy 1; p-JNK, phosphorylated c-Jun N-terminal kinase; *RIPK1*, receptor interacting protein kinase 1; *RIPK2*, receptor interacting protein kinase 2; *RIPK3*, receptor interacting protein kinase 3; ROS, reactive oxygen species; *SELENOI*, selenoprotein I; *SELENOO*, selenoprotein O; *SELENOS* or *SelS*, selenoprotein S; *SEPHS2*, selenophosphate synthetase 2; *SOCS3*, suppressor of cytokine signaling 3; SOD, superoxide dismutase; T3, triiodothyronine; T4, thyroxine; T-AOC, total antioxidant capability; TCR, T-cell receptor; *TNF-α*; tumor necrosis factor-α; *TR1*, thioredoxin 1; *TXNRD1*, thioredoxin reductase 1; VH, villous height; VH/CD, villous height/crypt depth; *ZO-1*, zonula occludens-1.

## Data Availability

The data presented in this study are available in this article.
